# Reply to “Letter to the Editor on Yurttas C et al. Limitations of laparoscopy to assess the peritoneal cancer index and eligibility for cytoreductive surgery with HIPEC in peritoneal metastasis”

**DOI:** 10.1007/s00423-023-02772-0

**Published:** 2023-01-14

**Authors:** Can Yurttas, Alfred Königsrainer, Philipp Horvath

**Affiliations:** Department of General, Visceral and Transplant Surgery, University Hospital Tübingen, Hoppe-Seyler-Str. 3, 72076 Tübingen, Germany

We have read the letter to the editor by Carboni and Valle on our work “Limitations of laparoscopy to assess the peritoneal cancer index and eligibility for cytoreductive surgery with HIPEC in peritoneal metastasis” with great interest. We are pleased with the initiated debate and appreciate that we have the opportunity to reply to the issues raised by the authors concerning doubts about the methodology and conclusions of our work.

Similar to one of our main conclusions, Carboni and Valle state that results from intermodal PCI assessment may be discrepant. We agree with their assumptions that the unusual long time between laparoscopic and open exploration in our cohort as well as the subjectivity and differing experience of surgeons might be explanatory for the PCI incongruencies observed in our study. A long time interval between laparoscopy and CRS is truly unfavorable but caused by limited surgical resources. Laparoscopy was mainly performed by surgeons involved in the field of CRS and HIPEC, so this argument can be refuted. Ideally, laparoscopic PCI should be assessed by the same surgeons that perform CRS and HIPEC. We indeed agree with the idea although it remains hypothetical if this actually holds true and it has to be proved by future trials. In any case, we already discussed these circumstances in our work and therefore advance the view that laparoscopy performed before premediated open exploration for CRS and HIPEC would be the ideal setup to rule out PCI discrepancies arising from subjectivity and possible tumor progression between open and laparoscopic PCI assessment.

Carboni and Valle further state that they disagree with the limitations of laparoscopy reported by us. In fact, from our data, we solely concluded that laparoscopy is, compared to open exploration, imprecise in the assessment of PCI comprising mainly underestimation thereby restricting its significance. The fact that laparoscopy may be deficient in evaluating the eligibility for complete cytoreduction is underlined by the persistently high rate of non-therapeutic laparotomies reported in the literature despite the implementation of laparoscopy [1–3, 5, 8]. In our discussion, however, we also oppose this restriction observed in our analysis to the overt advantages of laparoscopy in evaluating patients before open exploration for CRS and HIPEC. These include, among others, the preclusion of disseminated tumor extent and involvement of irresectable structures as well as the ability to obtain samples for histopathological examination [8].

We agree with the authors that our study would have been more informative if we had included the patients that were spared from open exploration due to laparoscopic findings. However, we set up our study to compare the PCI values subsequently assessed in patients that were scheduled for open exploration following laparoscopic PCI assessment. Thereby, patients that underwent immediate open exploration without prior laparoscopy or received chemotherapy in between both assessments were not included in our analysis. This is also true for gynecological patients that are exclusively treated at the department of gynecology and obstetrics in Tübingen, so that we were unable to include and systematically assess patients with ovarian or fallopian tube carcinoma.

Despite relevant limitations deriving from our retrospective study design and inhomogeneous and small study cohort that inevitably limits the informative value of our analysis, we carved out that PCI values assessed during laparoscopy do not necessarily equal those found during open exploration [8]. This, in part, might contribute to non-therapeutic laparotomies still occurring also in times of broadly accessible laparoscopy but must not mistakenly be interpreted as a general plea against laparoscopy.

We again thank the authors for their valuable comments made on our work. In agreement with Carboni and Valle as well as many other authors [1–7], we also consider laparoscopy a valuable tool and still utilize it in the management of patients with peritoneal metastasis at our center, but at the same time, we advocate for a more cautious interpretation of findings from laparoscopy.
